# Cross-talk between phosphatidic acid and ceramide during ethanol-induced apoptosis in astrocytes

**DOI:** 10.1186/1471-2210-5-3

**Published:** 2005-02-04

**Authors:** Beate Schatter, Shenchu Jin, Konrad Löffelholz, Jochen Klein

**Affiliations:** 1 Department of Pharmacology, School of Medicine, Johannes Gutenberg University of Mainz, Mainz, Germany; 2 Department of Pharmaceutical Sciences, School of Pharmacy, Texas Tech University Health Science Center, Amarillo, Texas, USA

## Abstract

**Background:**

Ethanol inhibits proliferation in astrocytes, an effect that was recently linked to the suppression of phosphatidic acid (PA) formation by phospholipase D (PLD). The present study investigates ethanol's effect on the induction of apoptosis in astrocytes and the formation of ceramide, an apoptotic signal. Evidence is presented that the formation of PA and ceramide may be reciprocally linked during ethanol exposure.

**Results:**

In cultured rat cortical astrocytes, ethanol (0.3–1 %, v/v) induced nuclear fragmentation and DNA laddering indicative of apoptosis. Concomitantly, in cells prelabeled with [^3^H]-serine, ethanol caused a dose-dependent, biphasic increase of the [^3^H]-ceramide/ [^3^H]-sphingomyelin ratio after 1 and 18 hours of incubation. As primary alcohols such as ethanol and 1-butanol were shown to inhibit the phospholipase D (PLD)-mediated formation of PA, a mitogenic lipid messenger, we tested their effects on ceramide formation. In astrocytes prelabeled with [^3^H]-serine, ethanol and 1-butanol, in contrast to t-butanol, significantly increased the formation of [^3^H]-ceramide. Moreover, exogenous PA, added to transiently permeabilized astrocytes, suppressed ethanol-induced [^3^H]-ceramide formation. Vice versa, addition of C_2_-ceramide to astrocytes inhibited PLD activity induced by serum or phorbol ester.

**Conclusion:**

We propose that the formation of ceramide in ethanol-exposed astrocytes is secondary to the disruption of phospholipase D signaling. Ethanol reduces the PA:ceramide ratio in fetal astrocytes, a mechanism which likely participates in ethanol-induced glial apoptosis during brain development.

## Background

The proliferation of astrocytes is stimulated by polypeptide growth factors such as PDGF, EGF, bFGF and IGF-1 acting on cellular signaling pathways which involve tyrosine kinases, protein kinase C, and the Ras-Raf-MAP kinase pathway [1,2]. Astroglial proliferation is also stimulated by neurotransmitters such as acetylcholine and glutamate [3,4], by direct stimulation of protein kinase C with phorbol ester [5,6], and by peptides such as endothelin and prolactin [7,8]. Astroglial proliferation is prominently inhibited by ethanol both in vivo and in vitro [9-11], and this interference likely contributes to the development of the fetal alcohol syndrome (alcoholic embryopathy) (reviewed in [12]). Ethanol has been shown to potently antagonize proliferative effects of several individual astroglial mitogens including PDGF, IGF-1, acetylcholine and prolactin [8,13-15].

The molecular target of ethanol's antimitogenic actions in astroyctes is not known with certainty, but inhibitory interactions of ethanol with lipid signaling pathways have been implicated [15]. Our group has recently reported strong evidence that the growth-inhibitory effect of ethanol in astrocytes is caused by the disruption of the phospholipase D (PLD) signaling pathway [16,17]. Under physiological conditions, PLD catalyzes the hydrolysis of phosphatidylcholine (PC) to yield phosphatidic acid (PA) and choline. In the presence of ethanol, however, PLD forms phosphatidylethanol (PEth), a non-physiological phospholipid, at the expense of PA. This PLD-specific phenomenon of transphosphatidylation is the reason why downstream events mediated by PLD activation and PA formation are dose-dependently inhibited in the presence of ethanol (or other primary alcohols such as 1-butanol).

In our previous work, we have found that astroglial PLD is activated by mitogenic factors including fetal calf serum (FCS), PDGF, and phorbol ester, and we observed that ethanol reduced both astroglial proliferation and PA formation in a parallel manner. 1-butanol reduced PA formation and DNA synthesis with the same potency while t-butanol was inactive for both effects [16]. More recently, we demonstrated that exogenous PLD as well as PA, when introduced into the cytosol by transient permeabilization, stimulated astroglial cell proliferation. Importantly, the action of PLD was suppressed in the presence of ethanol (0.3 %, v/v) while the mitogenic effect of PA was not affected [17]. Thus, disruption of the PLD signaling pathway by ethanol is sufficient to suppress astroglial cell proliferation.

Recent findings from other groups are also compatible with a central role for the PLD signaling pathway in ethanol toxicity in astrocytes. First, several mitogenic factors including those that are known to be particularly sensitive to ethanol activate PLD activity in astrocytes. This holds true for PDGF [16], acetylcholine [5,18], glutamate [19], phorbol esters [5,6], endothelin [20,21], and prolactin [22]. In fact, disruption of PLD signaling by ethanol was recently found to be responsible for ethanol's inhibitory effect on astroglial DNA synthesis induced by muscarinic agonist [23]. Second, PLD is activated via the mitogenic Ras-Ral pathway in many cell types [24], and PA, the immediate product of PLD activity, interacts with and activates proteins such as Raf kinase, protein kinase Cζ, and mTOR which are known to be central to mitogenic signaling (reviewed in [25,26]). In addition, PA is a precursor of diacylglycerol (DAG), the endogenous activator of classical PKC's, and of *lyso*-PA, a potent mitogen in many cell types [25,26]. Taken together, current evidence suggests that intact PLD signaling is a prerequisite for the proliferative effects of several mitogens, and that disruption of the PLD pathway by ethanol may be a common theme in ethanol-induced inhibition of astroglial proliferation.

The present study in fetal astrocytes was motivated by recent reports that ethanol induces apoptosis in astrocytes, an effect that was accompanied by activation of the sphingomyelinase pathway and formation of ceramide [27,28]. Apoptosis denotes an active cellular program causing cellular death upon contact with toxicants. Apoptotic cell death in the CNS has been under intensive study in recent years and involves several intracellular reaction cascades linked by the activation of caspases (reviewed in [29]). Apoptosis is almost universally accompanied by the formation of ceramide which may occur through *de novo*-synthesis, inhibition of ceramide breakdown, or activation of (acidic and/or neutral) sphingomyelinase (SMase), an enzyme which catalyzes the hydrolysis of sphingomyelin to ceramide and phosphocholine (see [30] for review). Ceramide has emerged as a second messenger for apoptotic pathways targeting kinases and phosphatases which are required for the execution of apoptotic cell death (reviewed in [31,32]). In cerebellar astrocytes and in glioma cells, ceramide levels were found to be reciprocally related to cell proliferation [33,34]. For the present study, we developed the hypothesis that the formation of ceramide may be secondary to the inhibition of PLD signaling which we had described earlier (see above). We now report that ethanol-induced ceramide formation in astrocytes is mimicked by 1-butanol, but not by t-butanol, and that PA, the product of PLD activity, antagonizes ethanol-induced formation of ceramide. We also found that ceramide is a potent inhibitor of stimulated PLD activity. Thus, we obtained evidence of a cross-talk between PA and ceramide, two lipid messengers with opposite effects on cellular proliferation.

## Results

### Markers of apoptosis

**Figure 1:**
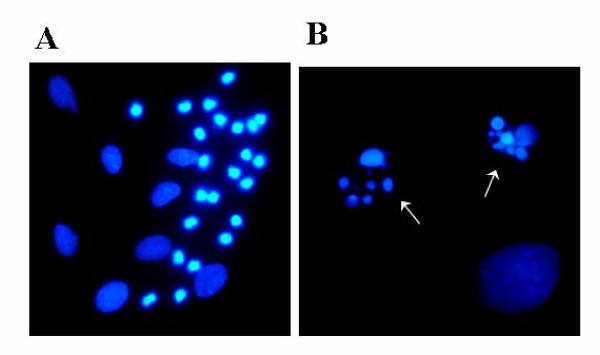
Nuclear fragmentation of astrocytes after treatment with ethanol. In this experiment, astrocytes were incubated with ethanol (1 %, v/v) for 21 hours, fixed in methanol/acetic acid (3:1) and stained with bisbenzimide (Hoechst 33258, 1 μg/ml). The characteristic condensation and fragmentation of nuclei indicates apoptosis. Enlargement: left picture, 400 fold; right picture, 1,000 fold. The experiment was repeated three times with similar results.

**Figure 2:**
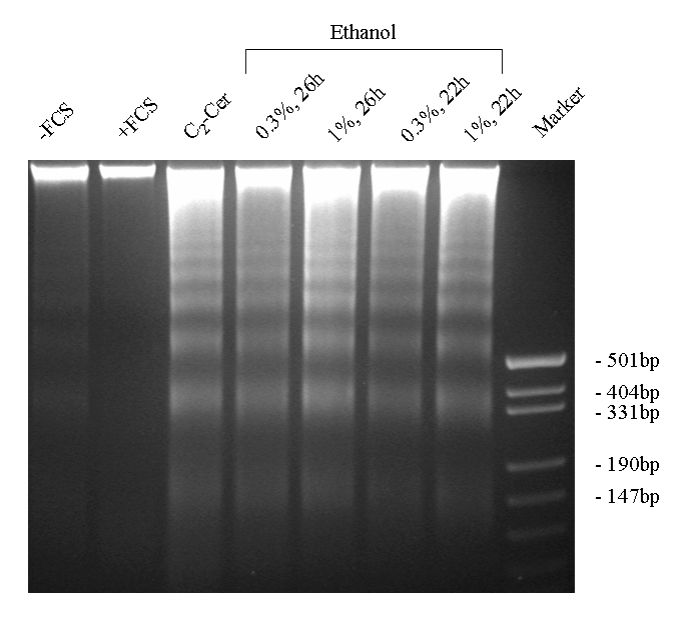
DNA fragmentation in astrocytes after treatment with ethanol. Astrocytes were incubated with the compounds given: lane 1, control (serum-free medium); lane 2, serum-containing medium; lane 3, C_2_-ceramide (50 μM) in serum-free medium; lanes 4–7, ethanol in serum-free medium (concentrations and times as given); lane 8, size markers. After incubation, cells were lysed, DNA was purified, separated on a 3 % agarose gel and stained with ethidium bromide. The experiment was repeated three times with identical results.

When primary astrocyte cultures were exposed to ethanol (0.3–1%, v/v), staining of the cells with Hoechst 33258, a dye intercalating into DNA, revealed condensation and fragmentation of the nuclei which was visible after 16 hrs; the maximum effect was observed after 21 hours (Fig. 1A). Higher magnification demonstrated the presence of "apoptotic bodies" in the nuclei (Fig. 1B). A similar effect was observed after treatment of the cells with the well-known apoptogen, staurosporine (1 μM), or with C_2_-ceramide (50 μM) but not with t-butanol (1%, v/v) (not illustrated). In parallel experiments, ethanol caused fragmentation of nuclear DNA in serum-starved astrocytes which is reflected by "DNA laddering" on agarose electrophoresis (Fig. 2). Serum withdrawal alone was not effective while incubation with C_2_-ceramide (50 μM) mimicked the effect of ethanol. Ethanol at 0.3% (v/v) was almost as effective as 1 % (Fig. 2).

### Effects of ethanol on sphingomyelin hydrolysis

**Figure 3:**
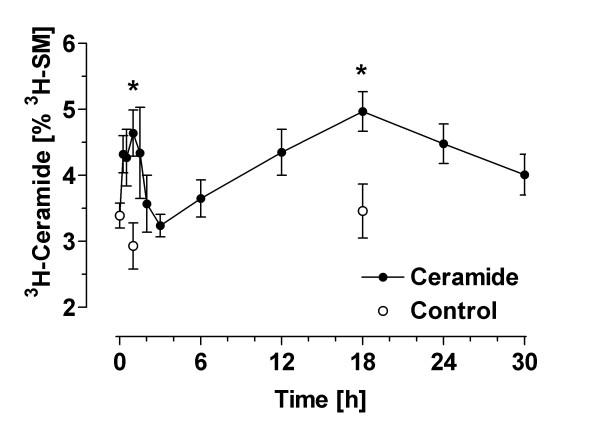
Formation of ceramide in ethanol-treated astrocytes: time course. Astrocytes were labeled with [^3^H]-serine for 72 hours, washed and treated with ethanol (0.3 %, v/v) in serum-free medium. At the indicated time points, the cells were extracted with methanol/chloroform (2:1), lipid extracts were separated by TLC, and radioactivity associated with [^3^H]-ceramide and [^3^H]-sphingomyelin was determined by liquid scintillation counting. Data (N = 3–7) are means ± S.E.M. and are expressed as [%] ceramide/sphingomyelin. Statistics: ANOVA, F_1,53 _= 2.28, p = 0.02. *, p < 0.05 vs. control at time zero (Dunnett's post test).

**Figure 4:**
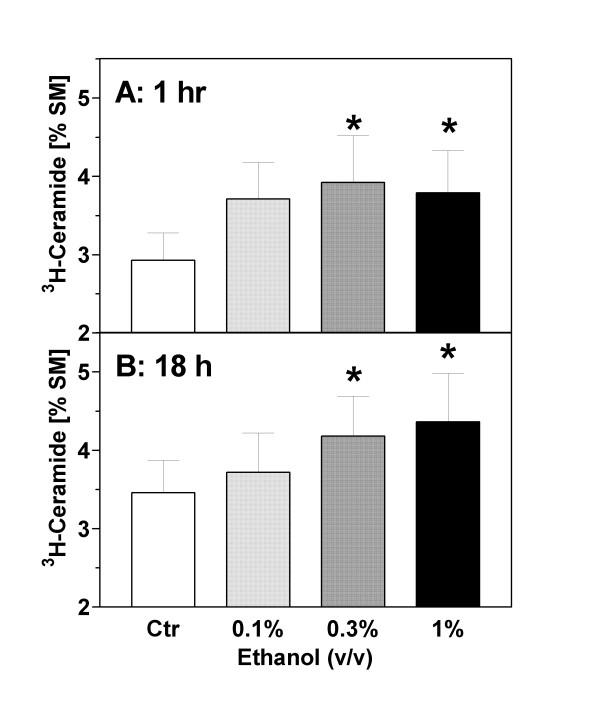
Formation of ceramide in ethanol-treated astrocytes: concentration dependence. Astrocytes were labeled with ^3^H-serine for 72 hours, washed and treated with ethanol (0.1–1 %, v/v). After **(A) **1 hour and **(B) **18 hours, the cells were extracted with methanol/chloroform (2:1), phospholipids were separated by TLC, and the radioactivity associated with ceramide and sphingomyelin was determined by liquid scintillation counting. Data (N = 5–6) are means ± S.E.M. and are expressed as [%] ceramide/sphingomyelin. Statistics: one-way ANOVA for repeated measurements, (A) F_3,19 _= 3.98, p = 0.03; (B) F_3,23 _= 4.88, p = 0.02. *, p < 0.05 vs. controls (Dunnett's post test).

Formation of [^3^H]-ceramide was measured after labeling sphingomyelin with [^3^H]serine. Under basal conditions, the ratio of [^3^H]-ceramide to [^3^H]-sphingomyelin ("C/S ratio") was approximately 1:30. This ratio was not significantly changed during serum withdrawal (Figs. 3 and 4). The incubation of astrocytes with ethanol (1 %, v/v) in serum-free medium caused an increase of [^3^H]-ceramide (cpm per dish) but did not significantly change the total labeling of the large pool of [^3^H]-sphingomyelin by [^3^H]-serine (data not shown). As the total incorporation of [^3^H]-serine into [^3^H]-sphingomyelin was somewhat variable between individual preparations, we used the C/S ratio to calculate ethanol-induced changes. As shown in Figs. 3 and 4, ethanol caused a significant increase of the astroglial C/S ratio in a biphasic and dose-dependent manner.

The rapid and transient phase of ceramide formation occurred within 15 min and reached a maximum at 1 hour after addition of ethanol (1 h value without ethanol: 2.93 ± 0.35%; 1 h value with 0.3% ethanol: 3.92 ± 0.60%; p = 0.02). A second increase gradually developed after 4 hours and reached a maximum at 18 hours of ethanol exposure (18 h value without ethanol: 3.46 ± 0.41 %; 18 h value with ethanol: 4.18 ± 0.51 %; p = 0.005). At this later time point, staurosporine (1 μM) caused an increase of the C/S ratio to 7.97 ± 1.78 % (p < 0.01; not illustrated).

### Inhibition of ceramide formation by PLD activity and phosphatidic acid

**Figure 5:**
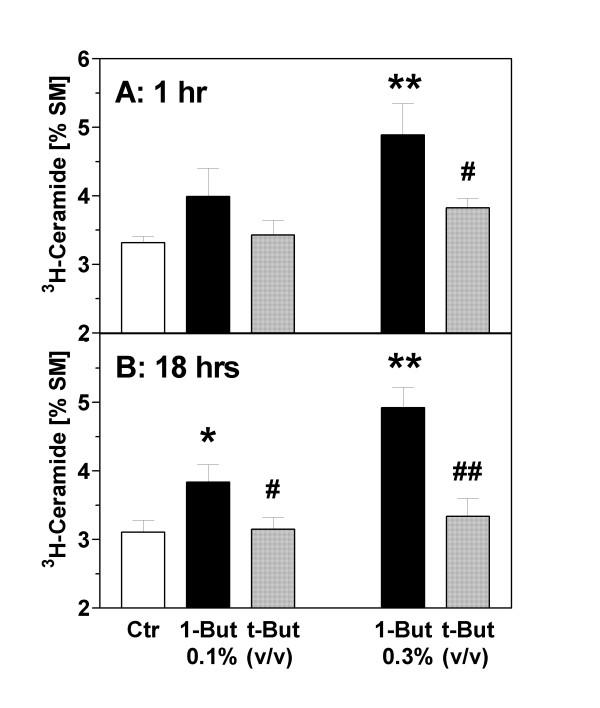
Effects of isomeric butanols on ceramide formation in astrocytes. Astrocytes were prelabeled with [^3^H]-serine for 72 hours, washed and treated with 1-butanol ("1-But") or t-butanol ("t-But"). After **(A) **1 hour and **(B) **18 hours, the cells were extracted with methanol/chloroform (2:1), phospholipids were separated by TLC, and the radioactivity associated with ceramide and sphingomyelin was determined by liquid scintillation counting. Data (N = 8–10) are means ± S.E.M. and are expressed as [%] ceramide/sphingomyelin. Statistics: Repeated measures ANOVA, (A) F_4,49 _= 7.2, p = 0.0002; (B) F_4,39 _= 25.0, p < 0.0001. *, p < 0.05; **, p < 0.01 vs. controls ("Ctr"). #, p < 0.05; ##, p < 0.01 vs. effect of 1-butanol (Tukey-Kramer multiple comparisons test).

To investigate whether ceramide formation is secondary to a disruption of PLD signaling, we used the isomeric alcohols, 1-butanol and t-butanol. 1-Butanol – but not t-butanol – is a substrate of PLD for transphosphatidylation and leads to the formation of phosphatidyl-1-butanol at the expense of PA. The correlations between butanol exposures and disruption of PLD signaling (i.e., suppression of PA formation) in astrocytes were documented in detail in our previous work [16]. In the present experiments we measured the differential effects of 1- and t-butanol on the formation of ceramide (C/S ratio) in astrocytes. As shown in Fig. 5, there was a tendency for an increased level of the C/S ratio after addition of 1-butanol at 0.1% while highly significant increases were observed with 0.3%. In contrast, t-butanol (0.1 and 0.3%) had no effect. This pattern was identical at the 1 hour and 18 hours time points (Fig. 5A and 5B). This pattern suggested a change of the C/S ratio that was secondary to the disruption of the PLD pathway.

**Figure 6:**
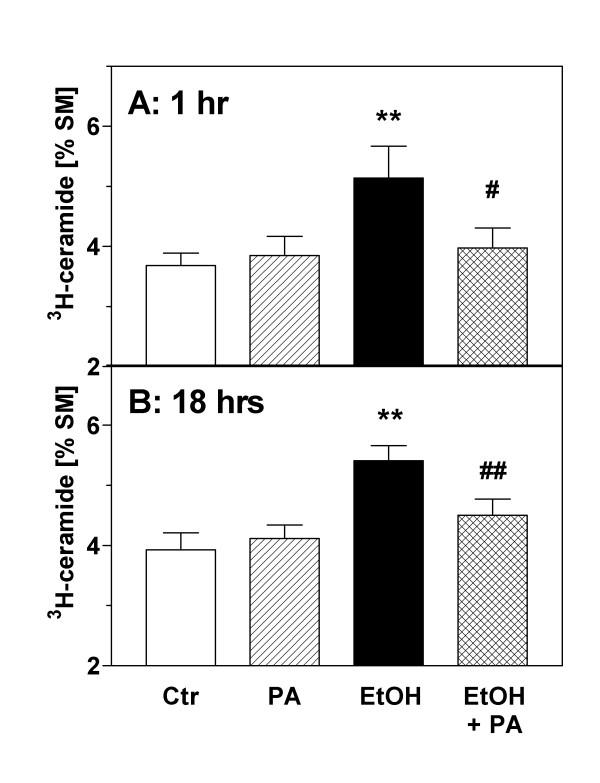
Effects of phosphatidic acid on ceramide formation in astrocytes. Astrocytes were prelabeled with [^3^H]-serine for 72 hours, washed and treated with PA (200 μM) or ethanol (EtOH, 0.3 % v/v) during transient permeabilization with streptolysin-O (144 ng/ml) in calcium-free medium. After 15 min, the cultures were washed and re-exposed to calcium-containing medium to initiate pore repair. After **(A) **1 hour and **(B) **18 hours, the cells were extracted with methanol/chloroform (2:1), phospholipids were separated by TLC, and the radioactivity associated with ceramide and sphingomyelin was determined by liquid scintillation counting. During the experiments, PA was only present for 15 min during cell permeabilization whereas ethanol was present throughout the incubation period. Data (N = 9) are means ± S.E.M. and are expressed as [%] ceramide/sphingomyelin. Statistics: Repeated measures ANOVA, (A) F_3,35 _= 5.52, p = 0.005; (B) F_3,35 _= 14.5, p < 0.0001. **, p < 0.01 vs. controls ("Ctr"). #, p < 0.05; ##, p < 0.01 vs. effect of ethanol (Tukey-Kramer multiple comparisons test).

To obtain more direct evidence for this hypothesis, we tested the influence of exogenous PA, the product of PLD, on the C/S ratio. For this purpose, we used a permeabilization procedure which makes use of an oxygen-insensitive mutant (C530A) of streptolysin-O (SL-O) to introduce the membrane-impermeable PA into the astroglial cytosol [17]. In preliminary experiments, we found that transient permeabilization with SL-O for 15 min by itself did not affect [^3^H]-ceramide levels (data not shown). Basal ceramide levels were also unchanged if exogenous PA was added to the astrocytes in the absence (data not shown) or presence of SL-O (Fig. 6). However, the ceramide formation evoked by ethanol (0.3 %) was significantly reduced in the presence of PA (Fig. 6). At the 1 h timepoint, the ethanol-induced effect (C/S ratio: 5.14 ± 0.53 %) was reduced by PA pretreatment to 3.98 ± 0.33 %, a relative reduction by 71 percent (Fig. 6A). At the 18 h timepoint, PA pretreatment reduced ethanol-induced ceramide formation by 61 percent (Fig. 6B).

### Inhibition of phospholipase D activity by ceramide

**Figure 7:**
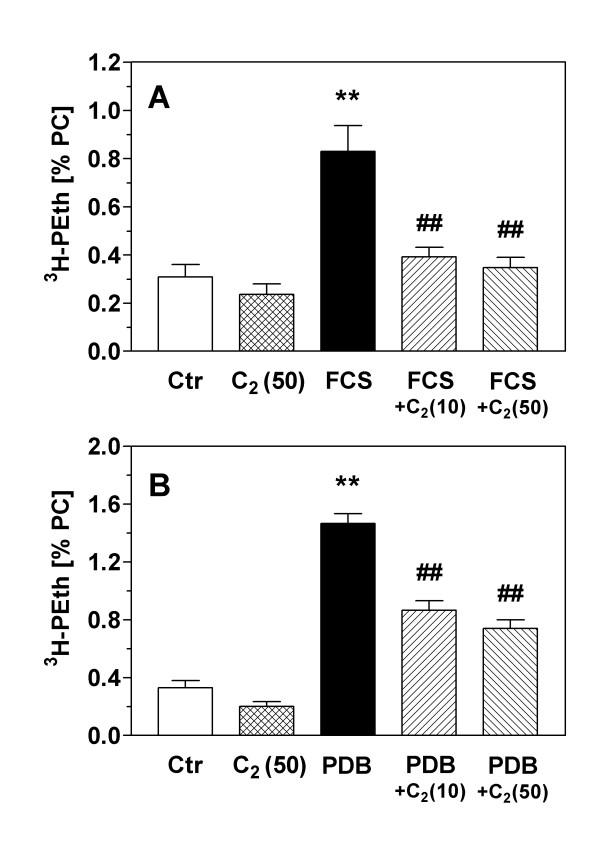
Inhibition of phospholipase D activity by ceramide. PLD activity in serum-starved, [^3^H]-glycerol-labeled astrocytes was determined by the transphosphatidylation assay; in the presence of ethanol, PLD converts [^3^H]-phosphatidylcholine (PC) into [^3^H]-phosphatidylethanol (PEth) which reflects PLD activity. In (A), PLD activity was stimulated by addition of medium containing 10% fetal calf serum (FCS) for 5 min. In (B), PLD activity was stimulated by 4β-phorbol-12β,13α-dibutyrate (PDB; 1 μM) for 30 min. C_2_-ceramide ("C_2_") was added to the cultures in concentrations of 10 and 50 μM 45 min before the addition of FCS and PDB, respectively. Data are means ± S.E.M. of 7–8 experiments and are expressed as [%] PEth/PC. Statistics: ANOVA, (A) F_4,36 _= 14.0, p < 0.0001; (B) F_4,37 _= 75.2, p < 0.0001. **, p < 0.01 vs. controls. ##, p < 0.01 vs. stimulated PLD activity (Tukey-Kramer multiple comparisons test).

As the previous experiments indicated an inhibitory effect of the PLD pathway on ceramide formation, the following experiment tested a possible effect of ceramide on PLD activity measured by the transphosphatidylation assay. In these experiments, the membrane-permeable C_2_-ceramide (50 μM) slightly but non-significantly reduced basal PLD activity by 30% (Fig. 7). However, when PLD activity was stimulated by addition of serum (Fig. 7A) or by PDB, a phorbol ester and stimulator of protein kinase C (Fig. 7B), C_2_-ceramide at both 10 and 50 μM strongly and significantly reduced PLD activity. Interestingly, serum-induced PLD stimulation was more sensitive to ceramide than PDB-induced PLD activity; the relative inhibitions for 10 and 50 μM C_2_-ceramide were (for serum stimulation) 84 and 93 % and (for PDB stimulation) 53 and 64 %, respectively.

## Discussion

We have previously documented that ethanol suppresses signaling through the mitogenic phospholipase D (PLD) pathway, and we and others have provided evidence that this effect may be responsible for the antiproliferative actions of ethanol in astrocytes [16,17,23]. The present study was motivated by findings that ethanol induces astroglial apoptosis via activation of the sphingomyelinase pathway [27,28]. We confirm these earlier reports by showing the induction of apoptotic markers and ceramide formation in ethanol-treated astrocytes. The novel finding of our study is that ethanol-induced formation of ceramide is reciprocally regulated by phosphatidic acid (PA) and the phospholipase D pathway which is itself inhibited by ceramide.

We used three different approaches to demonstrate that apoptotic cell death in astrocytes can be induced by exposure to ethanol. First, we report that ethanol can induce nuclear condensation and degradation (Fig. 1). Second, application of ethanol to serum-starved astroglial cultures caused "DNA laddering", a typical hallmark of apoptotic degradation of nuclear DNA (Fig. 2). The effect of ethanol was mimicked by a cell-permeable ceramide, C_2_-ceramide (Fig. 2), and by staurosporine (not illustrated). Third, ethanol induced an increase of ceramide in astroglial cultures (Figs. 3 and 4). Our findings clearly confirm that ethanol can induce apoptosis and ceramide formation in astrocytes, a finding which is in agreement with some [28] but not all [35] previous studies.

We performed a time course of [^3^H]-ceramide formation after ethanol exposure and found that it was maximal at 1 and 18 hours. Biphasic formations of ceramide such as those observed here have been described previously in a range of peripheral cell types although their significance is unclear; they may reflect different modes of ceramide formation, or different pools of ceramide [36]. At both time points of maximum ceramide formation (1 hour and 18 hours), we observed the same ethanol-evoked enhancement of ceramide formation. Importantly, apoptotic cell death and ceramide formation were induced by ethanol levels as low as 65 mM which corresponds to blood alcohol levels (0.3%) which are found in heavy drinkers.

It should be noted that the present experiments do not unequivocally identify the mechanism of ceramide formation. We used [^3^H]-serine to pre-label sphingomyelin for 72 hours, removed the precursor, and measured formation of ceramide as an increase of the [^3^H]-ceramide/ [^3^H]-sphingomyelin ratio during incubations with ethanol. This ratio most likely reflects the activity of sphingomyelinase(s), and sphingomyelinase activity was actually shown to be responsible for ethanol-induced ceramide formation in a recent study [28]. However, our present data do not exclude alternative pathways of increased ceramide formation such as *de novo*-synthesis of ceramide or inhibition of ceramidase.

The important findings of this study relate to the interaction between lipid second messenger pathways. It was known from previous work (see Introduction) that PA, the product of phospholipase D (PLD), mediates mitogenic stimulation in astrocytes whereas formation of ceramide by sphingomyelinase activation accompanies apoptosis. We now tested the hypothesis that ethanol causes astroglial apoptosis by inhibiting PLD and, as a consequence, stimulates the sphingomyelinase pathway. The results shown in Figs. 5 and 6 are evidence of a direct inhibitory influence of the PLD pathway on ceramide formation. First, we observed that the addition of 1-butanol, a primary alcohol which suppresses PLD signaling, caused an increase of ceramide levels (Fig. 5). This effect was not seen with the inactive isomer t-butanol which does not interfere with PLD signaling in astrocytes (see our previous study [16]). Second, we used transient permeabilization of astrocytes by streptolysin-O to introduce PA, the product of PLD, into the astroglial cytosol [17,37]. We found that exogenous PA almost completely prevented the ethanol-induced increase of ceramide at early (1 hr) and delayed (18 hrs) phases of ceramide formation (Fig. 6). The fact that ethanol and 1-butanol, but not t-butanol increase ceramide formation, whereas PA antagonized this effect, gives strong evidence that PLD-mediated formation of PA keeps ceramide levels low under basal conditions (Fig. 5), and that PLD activity antagonizes ceramide formation under the influence of toxicants (Fig. 6). Unfortunately, we could not determine the effect of exogenous PA on astroglial apoptosis because the permeabilization procedure was found to induce a delayed apopototic response in astrocytes (not shown).

We also probed the reciprocal effects of ceramide signaling on PLD activity. The results shown in Fig. 7 demonstrate that C_2_-ceramide inhibits PLD activity at a concentration as low as 10 μM. Basal PLD activity was only slightly inhibited, but the increases of PLD activity induced by addition of serum or phorbol ester were strongly antagonized. This finding in astrocytes corroborates previous reports that ceramide can inhibit PLD signaling in peripheral cell types [38,39]. It remains a matter of speculation why serum-induced PLD was somewhat more susceptible to inhibition by ceramide than phorbol ester-stimulated activity. Growth factors in serum and phorbol ester may affect different signaling pathways leading to PLD activation, and we previously presented inhibition data with bacterial toxins which supported this idea for astroglial PLD [40]. Interestingly, ethanol was also observed to inhibit serum- and growth factor-mediated astroglial proliferation more effectively than phorbol ester-induced proliferation [6,16]. At this time, we cannot distinguish which isoform of PLD is responsible for PA formation; previous attempts to selectively down-regulate astroglial PLDs failed due to the long biological half-lives of the proteins [41]. The molecular target of ceramide for inhibiting PLD also remains to be identified; previous work has implicated direct inhibition of PLD by ceramide as well as upstream molecules such as protein kinase C which activate PLD [42,43].

## Conclusions

**Figure 8:**
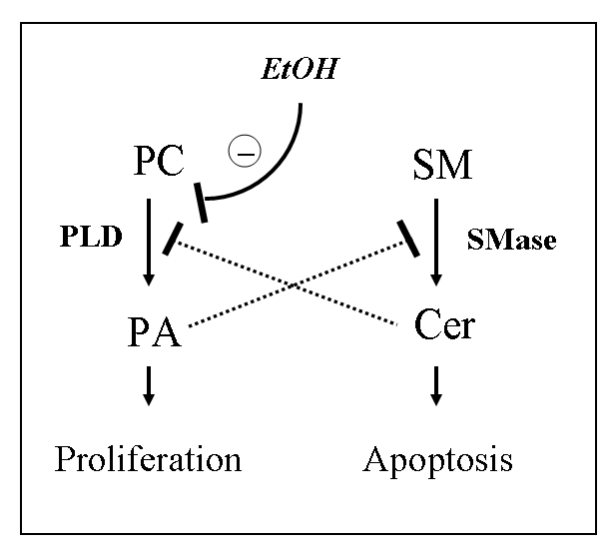
Hypothetical cross-talk between phospholipase D and sphingomyelin pathways in astrocytes, and effect of ethanol. Phosphatidic acid (PA), the product of phosphatidylcholine (PC) hydrolysis by phospholipase D (PLD), inhibits hydrolysis of sphingomyelin (SM) by sphingomyelinase (SMase). Vice versa, ceramide (Cer), the product of SM hydrolysis by SMase, inhibits activation of PLD. Ethanol induces apoptosis by disrupting the mitogenic PLD signaling pathway thereby decreasing the PA:Cer ratio and disinhibiting the pro-apoptotic SMase pathway.

In summary, the present results give evidence of a cross-talk between lipid-signaling pathways in astrocytes such that the product of PLD, namely PA, inhibits ceramide formation whereas ceramide inhibits PLD activation (Fig. 8). The experimental evidence suggests that the ratio "PA:ceramide" contributes to the decision whether astrocytes proliferate or undergo apoptosis. Our data suggest that ethanol induces astroglial apoptosis during brain development by disrupting PLD signaling, thereby reducing PA and increasing ceramide formation. This effect likely contributes to the microencephaly and delay of brain development observed in fetal alcohol syndrome.

## Methods

### Materials

[^3^H]-Serine and [^3^H]-glycerol were from Biotrend (Köln, Germany). Ceramide (from bovine brain), C_2_-ceramide (N-acetyl-D-*erythro*-sphingosine), and staurosporine were from Alexis (Lausen, Switzerland). Hoechst 33258, L-α-phosphatidic acid (sodium salt, from egg yolk) and 4β-phorbol-12β,13α-dibutyrate (PDB) were from Sigma (Deisenhofen, Germany); most other chemicals and TLC plates were obtained from Merck (Darmstadt, Germany) or Roth (Karlsruhe, Germany) at the highest purity available. Fetal calf serum (South America) was from Invitrogen, cell culture materials were from Sarstedt (Nürnbrecht, Germany). Phosphatidylethanol (PEth) standard was synthesized as described [16]. Recombinant, oxygen-insensitive streptolysin-O (C530A) was prepared as described [44].

### Cell culture

Astrocyte-rich cultures were prepared from newborn rat pups. Cerebral hemispheres were collected, meninges and blood vessels were removed, the brain tissue was dissociated by trituration, passed through a 50 μm nylon mesh, and the cells were seeded onto plastic culture dishes (14,000 cells per cm^2^). The growth medium was DMEM containing 10% fetal calf serum (FCS), 2 g/l NaHCO_3_, 100 U/ml penicillin and 100 μg/ml streptomycin. The cells were incubated at 37°C in a 95/5% mixture of air and carbon dioxide. For the experiments, astrocytes were grown for two weeks in culture and were used when they reached confluency. As judged by GFAP immunostaining, these cultures contained >90% astrocytes.

### Fluorescence microscopy

Cells were seeded on microplates (5,000 cells per 200 μl) and incubated with different apoptogens (ethanol 0.3 and 1 %, C_2_-ceramide 50 μM or staurosporine 1 μM) for 24 hrs in serum-free medium. Subsequently, cells were washed and fixed with ice-cold methanol/acetic acid (3:1). Dried and re-hydrated cells were stained with bisbenzimide (Hoechst 33258, 1 μg/ml) solution for 10 min, washed and sealed in gelatin. Photographs were obatined using a Leica Leitz DMRB fluorescence microscope and a Nikon Digital Camera DXM.

### DNA fragmentation

The test was carried out as described [45] with some modifications. Briefly, cells were incubated with apoptogens (Ethanol 0,3%, 1%; C2-Ceramide 50 μM) in serum-free medium. Then, the cells were transferred and resuspended in Tris-EDTA buffer containing 0.5% Igepal CA 630. Further lysis was performed in buffer containing RNAse A (100 μg/μl), proteinase K (0,5 μg/ml) and SDS (1.2 %). After 5 minutes, the clear solution was mixed with 3 M CsCl in acetate buffer. Precipitated debris and chromosomal DNA was removed by centrifugation, and the supernatant was loaded onto a QIAprep column (twice), centrifuged and eluted by hypotonic Tris-EDTA buffer. The eluate was analyzed by electrophoresis on a 3 % agarose gel and visualized with ethidium bromide.

### Measurement of [^3^H]-ceramide formation

Phospholipids were labeled by addition of [^3^H]-serine (1 μCi/ml) to astrocytes kept in growth medium (DMEM plus FCS) for 72 h. After washing, cells were incubated in serum-free medium with ethanol, butanol, C_2_-ceramide or staurosporine (see Results). C_2_-ceramide or staurosporine were added in DMSO; the final DMSO concentration was < 0.1 %. At the end of the incubation, cells were fixed with methanol, transferred and extracted first with with chloroform: methanol (1:1), then with chloroform: methanol: water (10:10:9) to separate water and lipid phases. After addition of ceramide and sphingomyelin standards, the lower (lipid) phase was evaporated, taken up in chloroform:methanol (3:2) and separated by thin-layer chromatography (TLC). For the determination of [^3^H]-ceramide, we used one-dimensional TLC (HP-TLC plates Merck 11845; eluent: chloroform/acetic acid 9:1). For the determination of [^3^H]-sphingomyelin, we used two-dimensional TLC (TLC plates Merck 1.05721); solvent I was chloroform/methanol/25% aqueous ammonia (13:7:1), solvent II was chloroform/ methanol/water/acetic acid (30:30:2:5). ^3^H-lipids were visualized by using iodine vapor, spots were scraped and suspended in scintillation cocktail (Lumasafe Plus), and radioactivity was measured by liquid scintillation counting (Packard 1600 CA). Formation of [^3^H]-ceramide was calculated as percentage of radioactivity found in [^3^H]-sphingomyelin.

### Cell permeabilization

For the introduction of PA, astrocytes were transiently permeabilized with streptolysin-O [37]. Briefly, astrocytes were washed and exposed to an oxygen-insensitive mutant of streptolysin-O (C530A) in calcium-free HBSS buffer (prepared by dissolving 8 g NaCl, 0.4 g KCl, 60 mg KH_2_PO_4_, 60 mg Na_2_HPO_4 _× 2 H_2_O, 100 mg glucose in 1 L of sterile water). PA (sodium salt; 200 μM) was added as a suspension in buffer prepared by sonication. After 15 min, the cells were washed and incubated in serum-free, calcium-containing DMEM. Formation of [^3^H]-ceramide was determined as described above. Ethanol, if present, was present throughout the incubation period. Using 144 ng/ml of streptolysin-O, this procedure yielded transient permeabilization of > 80% of astrocytes followed by repair of the pore in > 80% of permeabilized cells. The procedure was previously found to allow the entry of approx. 10^6 ^molecules of PA per cell [17].

### Determination of phospholipase D activity

Phospholipase D activity was determined using the transphosphatidylation assay [46]. For this purpose, astrocytes were kept in serum-free medium containing [^3^H]-glycerol (1 μCi/ml) for 24 h in order to label phospholipids. More than 60% of the phospholipid label was associated with phosphatidylcholine (not illustrated). Subsequently, the cells were washed and re-exposed to medium containing ethanol (2 %) and PDB (1.0 μM) or FCS (10 %, v/v) as stimulators. PDB and C_2_-ceramide, when used, were dissolved in DMSO (end concentration of DMSO < 0.1 %). After 30 min of reaction time, the cells were washed in cold phosphate-buffered saline and extracted as described above for ceramide determinations. After addition of phosphatidylethanol (PEth) and PA standards, aliquots of the lipid phase were separated by two-dimensional TLC (TLC plate Merck 1.05721) using chloroform/methanol/25 % aqueous ammonia (13:7:1) for the first run and the upper phase of ethylacetate/isooctane/ acetic acid/water (13:2:3:10) for the second run. Individual phospholipids were stained by iodine, and the spots corresponding to PEth, PA and phosphatidylcholine (PC) were isolated and counted for radioactivity in a scintillation counter. To determine PLD activity, formation of [^3^H]- PEth was calculated as percentage of [^3^H]-PC.

### Statistics

Data are shown as means ± SEM of N experiments whereby N refers to the number of different astroglial preparations from different animals. Results were obtained from two replicate dishes which were pooled to represent a single experiment. Statistical calculations were performed by GraphPad InStat 3.0 program package, using analysis of variance (ANOVA) of paired or unpaired data as indicated in text and figure legends.

## Abbreviations

Cer, ceramide; FAS, fetal alcohol syndrome; FCS, fetal calf serum; PA, phosphatidic acid; PC, phosphatidylcholine; PDB, 4β-phorbol-12β,13α-dibutyrate; PEth, phosphatidylethanol; PKC, protein kinase C; PLD, phospholipase D; SL-O, streptolysin-O; SM, sphingomyelin.

## Author's contributions

B.S. carried out the cell culture experiments and phospholipid measurements and participated in the experiments concerning apoptotic markers. S.J. contributed substantially to the apoptotic marker experiments. K.L. participated in the design of the study and the final draft of the manuscript. J.K. conceived and supervised the study, performed the statistical analyses and drafted the manuscript.
